# Enhancing preoperative diagnosis of microvascular invasion in hepatocellular carcinoma: domain-adaptation fusion of multi-phase CT images

**DOI:** 10.3389/fonc.2024.1332188

**Published:** 2024-01-25

**Authors:** Zhaole Yu, Yu Liu, Xisheng Dai, Enming Cui, Jin Cui, Changyi Ma

**Affiliations:** ^1^ School of Automation, Guangxi University of Science and Technology, Liuzhou, Guangxi, China; ^2^ Laboratory of Artificial Intelligence of Biomedicine, Guilin University of Aerospace Technology, Guilin, Guangxi, China; ^3^ Department of Radiology, Jiangmen Central Hospital, Jiangmen, Guangdong, China

**Keywords:** hepatocellular carcinoma, microvascular invasion, multi-modal, domain adaptation, feature fusion

## Abstract

**Objectives:**

In patients with hepatocellular carcinoma (HCC), accurately predicting the preoperative microvascular invasion (MVI) status is crucial for improving survival rates. This study proposes a multi-modal domain-adaptive fusion model based on deep learning methods to predict the preoperative MVI status in HCC.

**Materials and methods:**

From January 2008 to May 2022, we collected 163 cases of HCC from our institution and 42 cases from another medical facility, with each case including Computed Tomography (CT) images from the pre-contrast phase (PCP), arterial phase (AP), and portal venous phase (PVP). We divided our institution’s dataset (n=163) into training (n=119) and test sets (n=44) in an approximate 7:3 ratio. Additionally, we included cases from another institution (n=42) as an external validation set (test1 set). We constructed three single-modality models, a simple concatenated multi-modal model, two current state-of-the-art image fusion model and a multi-modal domain-adaptive fusion model (M-DAFM) based on deep learning methods. We evaluated and analyzed the performance of these constructed models in predicting preoperative MVI using the area under the receiver operating characteristic curve (AUC), decision curve analysis (DCA), and net reclassification improvement (NRI) methods.

**Results:**

In comparison with all models, M-DAFM achieved the highest AUC values across the three datasets (0.8013 for the training set, 0.7839 for the test set, and 0.7454 for the test1 set). Notably, in the test set, M-DAFM’s Decision Curve Analysis (DCA) curves consistently demonstrated favorable or optimal net benefits within the 0-0.65 threshold probability range. Additionally, the Net Reclassification Improvement (NRI) values between M-DAFM and the three single-modal models, as well as the simple concatenation model, were all greater than 0 (all p < 0.05). Similarly, the NRI values between M-DAFM and the two current state-of-the-art image fusion models were also greater than 0. These findings collectively indicate that M-DAFM effectively integrates valuable information from multi-phase CT images, thereby enhancing the model’s preoperative predictive performance for MVI.

**Conclusion:**

The M-DAFM proposed in this study presents an innovative approach to improve the preoperative predictive performance of MVI.

## 1 Introduction

Microvascular invasion (MVI) is one of the significant factors contributing to postoperative recurrence of hepatocellular carcinoma (HCC) (1–4), exerting a pronounced impact on disease recurrence and shortened survival in HCC patients (5–7). When MVI is positive in cases of HCC, the short-term recurrence rate of small liver cancers (8) (liver cell tumors <2cm) is higher, and patients with liver cell tumors ≥2cm exhibit lower long-term survival rates (9). Therefore, MVI is commonly regarded as a marker to assess the malignancy degree of HCC (10). However, in clinical practice, the presence of MVI can only be confirmed through histopathological examination of resected tumor tissue postoperatively (11, 12). Accurately predicting the preoperative MVI status in a noninvasive manner remains a challenge.

Prior research has demonstrated the feasibility of preoperative MVI prediction in HCC using Computed Tomography (CT) images (13), and many studies have extracted radiological features from CT images to construct radiological models for predicting the preoperative MVI status (2, 14, 15). Since the extraction of radiological features relies on the subjective expertise of radiologists, less experienced radiologists may overlook valuable features (16). Additionally, radiological features are often considered low to mid-level features, which may not fully capture the heterogeneity of HCC (17).

Deep learning based on Convolutional Neural Networks (CNN) has the capacity to automatically extract high-level features relevant to the target problem in CT images, surpassing explicitly designed low and mid-level features (18–21). Research has indicated that deep learning methods exhibit excellent performance in differentiating liver lesions and classifying fibrosis, offering diagnostic accuracy comparable to pathological gold standards (22, 23). In previous studies, deep learning methods have been applied to predict the preoperative status of MVI. For example, Liu et al. (24) used AP-phase CT images to construct a deep learning model and combined it with clinical factors for preoperative MVI prediction. Jiang et al. (25), on the other hand, built deep models using arterial phase (AP), portal venous phase (PVP), and delayed phase (DP) CT images separately and concatenated the deep features from these three phases to predict the preoperative MVI status. While these studies have achieved certain effectiveness in preoperative MVI prediction, they also exhibit certain limitations. For instance, Liu et al. used only a single-phase CT image, limiting their ability to comprehensively evaluate tumor characteristics. Jiang et al., although combining information from different phases of CT images, did not address the issue of feature distribution differences during the fusion process.

To address these issues, our study proposes a multimodal domain-adaptive fusion model based on deep learning. This model employs deep learning methods to extract information from CT images acquired at different phases, enabling a more comprehensive evaluation of HCC characteristics. Furthermore, it employs domain adaptation to align the feature distributions of various CT images, thereby enhancing the quality of the fused features. To the best of our knowledge, there is limited research considering the differences in data distribution between different modalities when utilizing multimodal image information. Our study aims to investigate the effectiveness of the domain-adaptive fusion method for preoperative MVI prediction in HCC, in comparison to single-modal and multimodal simple concatenation methods. Our research provides a novel approach to effectively integrate multimodal image information for predicting the preoperative MVI status.

## 2 Materials and methods

**Figure 1:**
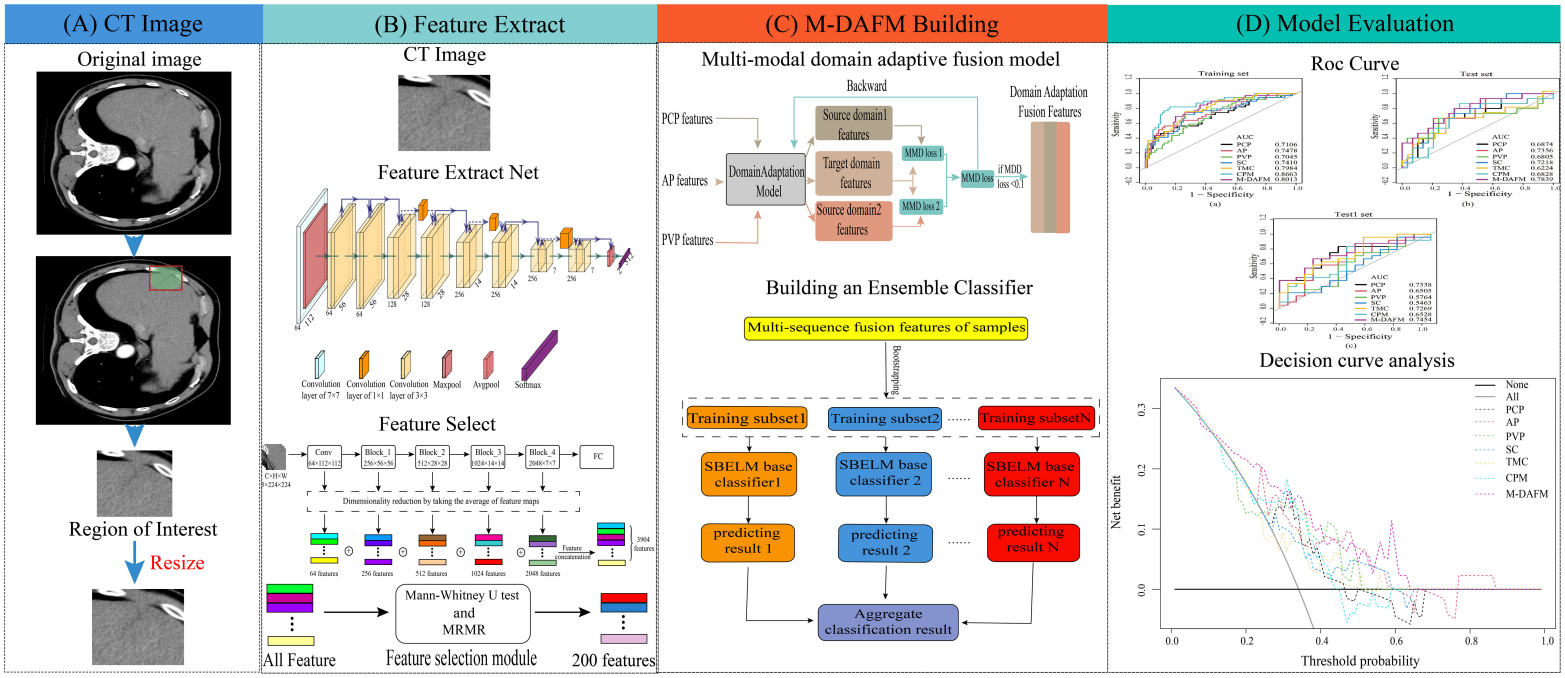
The overall design flowchart. **(A)** ROI extraction process; **(B)** DL feature extraction process; **(C)** M-DAFM building process; **(D)** Model evaluation.

The ethics committee of our hospital has granted approval for this retrospective study. Since the data is sourced from an existing institution and imposes no additional burden on the patients, the requirement for informed consent has been waived. **Figure 1** provides a schematic representation of the study’s design.

### 2.1 Patients

We conducted a retrospective study by querying our medical institution’s pathology database from January 2008 to May 2022 to identify patients who underwent hepatic resection surgery for HCC. The patient data collected by our institution predominantly employs major resection as the types of resection. The inclusion criteria for our study were as follows: (a) patients who did not receive any other anti-tumor treatments before surgery (including liver resection, liver transplantation, chemotherapy, radiation therapy, radiofrequency ablation, immunosuppressive therapy); (b) liver nodules with comprehensive histopathological descriptions in the pathology reports; (c) a time interval of no more than 4 weeks between preoperative CT examination [including pre-contrast phase (PCP), arterial phase (AP), portal venous phase (PVP)] and surgery. We excluded HCC patients with pathological results obtained through puncture and patients with artifacts in imaging and incomplete clinical information. A total of 163 patients with CT images from our institution met the inclusion and exclusion criteria. Subsequently, we randomly divided this dataset into a training set (n=119) and a test set (n=44) in an approximate ratio of 7:3. Statistical analysis revealed that in the training set, the rate of MVI was found to be 32.8% (39/119). Furthermore, we gathered 42 cases from external medical institutions to constitute an external validation set (test1 set). These cases adhere to the inclusion and exclusion criteria of our institution, and undergo the same preprocessing procedures as our institution’s pathology. This was done to further assess the predictive performance of the model on previously unseen data. The inclusion and exclusion criteria of our medical institution are presented in Electronic **Supplementary Material S1**.

### 2.2 Medical history and laboratory parameters

Age, gender, hepatocirrhosis status, and the presence of hepatitis B surface antigen (HBsAg) were documented for every patient. A range of serum biochemical parameters related to liver function were assessed for each patient within two weeks before or after the CT examination. These parameters encompassed α-fetoprotein (AFP), Carbohydrate antigen 199 (CA199), total bilirubin (TBIL), direct bilirubin (DBIL), indirect bilirubin (IBIL), alanine aminotransferase (ALT), aspartate aminotransaminase (AST), albumin, total protein, alkaline phosphatase (ALP), and platelet count (PLT). The baseline characteristics of the included cohorts are summarized in the **Table 1**.

**Table 1:** Baseline characteristics of the CT training set and test set.

CT Dataset						
Characteristics	Training (n=119)			Test (n=44)		
	MVI negative (n=80)	MVI positive (n=39)	p Value	MVI negative (n=29)	MVI positive (n=15)	p Value
Age (years)	56.26 ± 10.86	55.90 ± 12.08	0.869	58.00 ± 8.30	52.60 ± 9.21	0.055
Gender (n)			0.364			0.319
Male	62 (77.5%)	33 (84.6%)		27 (93.1%)	12 (80%)	
Female	18 (22.5%)	6 (15.4%)		2 (6.9%)	3 (20%)	
HBsAg status			0.605			0.171
Negative	21 (26.2%)	12 (30.8%)		10 (34.4%)	2 (13.3%)	
Positive	59 (73.8%)	27 (69.2%)		19 (65.6%)	13 (86.7%)	
Hepatocirrhosis status			0.647			1.000
Absent	28 (35%)	12 (30.7%)		7 (24.1%)	4 (26.6%)	
Present	52 (65%)	27 (69.3%)		22 (75.8%)	11 (73.4%)	
Log10AFP	3.21 ± 3.85	3.80 ± 4.25	0.044*	2.78 ± 3.43	3.55 ± 3.97	0.117
CA199	36.58 ± 62.24	80.60 ± 94.72	0.003*	22.52 ± 18.95	25.45 ± 22.26	0.650
TBIL	20.05 ± 12.57	14.28 ± 12.66	0.021*	19.94 ± 10.71	17.96 ± 8.10	0.534
DBIL	8.95 ± 8.32	6.67 ± 9.29	0.178	9.00 ± 4.78	8.20 ± 4.86	0.605
IBIL	11.10 ± 5.58	7.72 ± 4.58	0.001*	10.95 ± 6.60	9.76 ± 3.85	0.525
ALT	182.10 ± 367.57	101.70 ± 87.90	0.181	213.65 ± 248.3	328.54 ± 384.18	0.236
AST	134.23 ± 161.64	106.26 ± 85.78	0.314	171.33 ± 172.80	33.97 ± 373.67	0.046
Albumin	35.72 ± 5.73	33.22 ± 5.49	0.026*	33.63 ± 4.76	32.53 ± 3.77	0.439
Total protein	62.19 ± 8.68	59.02 ± 8.64	0.063	59.55 ± 8.07	55.12 ± 7.01	0.079
ALP	100.21 ± 42.96	119.01 ± 59.68	0.052	101.10 ± 69.79	197.20 ± 422.91	0.235
PLT	169.19 ± 56.65	178.05 ± 72.87	0.468	155.23 ± 91.47	199.40 ± 87.27	0.131

AFP, α-fetoprotein; CA199, Carbohydrate antigen 199; TBIL, total bilirubin; DBIL, direct bilirubin; IBIL, indirect bilirubin; ALT, alanine aminotransferase; AST, aspartate aminotransaminase; ALP, alkaline phosphatase; PLT, platelet count. * P < 0.05 indicates statistical significance; The numbers following ± represent the standard deviation.

### 2.3 Imaging scans

The CT scanning devices used in this study were the 16-detector CT (SOMATOM Sensation 16, Siemens Healthineers), the 64-detector CT (Aquilion 64, Canon Medical Systems), and the dual-source CT (SOMATOM Force, Siemens Healthineers). Patients maintained a supine position and held their breath during the procedure. The scanning sequences consisted of the pre-contrast phase (PCP), the arterial phase (AP, 30 seconds after contrast injection), and the portal venous phase (PVP, 60-70 seconds after contrast injection). The CT parameters included: tube voltage set at 120 kV, effective tube current-exposure time product ranging from 200 to 350 mAs, matrix size of 512×512, and a slice thickness of either 1.0 or 3.0 mm.

### 2.4 Radiologist assessment

The study utilized three different-phase CT images: PCP, AP, and PVP. Two radiologists, each possessing more than 5 and 11 years of expertise in abdominal imaging, independently conducted image assessments. These assessments were carried out in isolation from each other, with no knowledge of their respective ratings and no access to pathological findings. The degree of their confidence in detecting MVI was documented using a 5-point scale: 5, indicating a definite positive diagnosis; 4, signifying a probable positive finding; 3, expressing uncertainty; 2, suggesting a potential negative result; and 1, denoting a definite negative assessment (12). In cases of discordance, these two radiologists held discussions to reach a consensus score.

The summary of radiological features encompassed the following criteria: (1) tumor diameter (<5cm = 0; ≥5cm = 1); (2) the number of tumors (<2 = 0; ≥2 = 1); (3) the presence of a pseudocapsule (absent = 0; present = 1); (4) intratumoral necrosis (absent = 0; present = 1); (5) intratumoral hemorrhage (absent = 0; present = 1); (6) peritumoral enhancement during the arterial phase (AP) (absent = 0; present = 1); (7) AP hyperenhancement (absent = 0; present = 1); (8) wash-in and wash-out patterns (absent = 0; present = 1). Scores equal to or greater than 4 signified a heightened likelihood of MVI presence. Each image was individually examined and rated. In cases of multiple lesions, the option of surgical resection was considered.

### 2.5 Pathological diagnosis

The reference criteria for identifying MVI relied on the pathological examination of surgical specimens. MVI was specifically characterized as the presence of a tumor within a vascular space lined with endothelial cells, as visualized under microscopy (26). Moreover, to ensure precision, all our pathological findings underwent thorough review by a pathologist with twelve years of experience.

### 2.6 Tumor segmentation

After extracting patient images from our institutional picture archiving and communication system (PACS), we perform image de-identification and store them in the NIfTI format. Subsequently, these images are imported into 3D-Slicer (version 5.0.2). Next, we proceed with delineating the region of interest (ROI) on the CT images for each phase separately. The entire tumor is outlined at three distinct layers: the initial layer, the section with the maximum cross-sectional area, and the final layer. To ensure comprehensive coverage of the tumor, an additional 1-centimeter extension is applied at the margins. This delineation process is semi-automated to save the effort of radiologists and reduce the interference of subjective factors. The flowchart for image segmentation and preprocessing is presented in Electronic **Supplementary Material S2**.

### 2.7 Building the multi-modal domain adaptive fusion model

Multi-modal domain adaptive fusion model (M-DAFM) utilizesa convolutional neural network to extract feature information from the target task. It can predict the occurrence of MVI in HCC within a given ROI without the need for precise lesion segmentation. The training process of M-DAFM in this study consists of three stages: first, deep learning models are employed to extract features from single-phase CT images; then, domain adaptation techniques (27) are applied to align the distributions of features among the single-phase CT images and fuse these features; finally, an ensemble sparse Bayesian extreme learning machine (ESBELM) is used for preoperative prediction of MVI status in HCC. Detailed parameters for training the deep learning model can be found in Electronic **Supplementary Material S3**.

In the feature extraction stage, we employ a pre-trained ResNet18 model on ImageNet to extract features from multiple single-phase CT images, including PCP, AP, and PVP. Each single-phase image yields 3904 features. For a comprehensive understanding of the deep feature extraction process, please refer to Electronic **Supplementary Material S4**.

In the domain adaptation feature fusion stage, we perform feature selection using Mann-Whitney U test (28) and Maximum Relevance Minimum Redundancy (MRMR) algorithm (29) on the features extracted from individual single-phase images, selecting the top 200 features most relevant to the target task. Domain adaptation is a learning paradigm within transfer learning that primarily addresses distributional differences between the target domain and the source domain, enabling the adaptation of the source domain distribution to the target domain. In clinical practice, CT images from the PCP, AP, and PVP phases typically reflect relevant information about tumors from different perspectives. Consequently, there are often distributional differences among them. To alleviate these differences, we employ domain adaptation methods, treating the AP phase features as the target domain and the PCP and PVP phase features as the source domain, the Maximum Mean Discrepancy (MMD) (30) is utilized as the loss function to quantify the distributional differences between the source and target domains. This alignment aims to ensure that PCP, AP and PVP features exhibit similar distributions. The domain adaptation fusion algorithm proposed in this article can be divided into three steps: 1) We select the AP phase CT image features as the target domain and the PCP and PVP phase CT image features as two source domains. The purpose is to use the target domain as a standard to make the data distribution of the source domains closer to the target domain. 2) We use maximum mean discrepancy (MMD) as the model’s loss function to measure the distribution difference between the source domain (PCP and PVP features) and the target domain (AP features). By training the model to reduce these distribution differences, we make the distribution of the source domain tend to be consistent with the target domain. 3) We use a feature concatenation strategy to fuse the distribution-consistent PCP, AP, and PVP features, aiming to improve the model’s performance on unknown datasets. For a detailed description of the feature fusion process, please refer to Electronic **Supplementary Material S5**.

In the classification stage, we construct an ESBELM classifier. This classifier incorporates Bayesian linear regression algorithms into the framework of extreme learning machines (31) to reduce feature dimensions and mitigate model overfitting. Additionally, the classifier enhances model classification performance through the ensemble of multiple base classifiers. Detailed information about classifier construction can be found in Electronic **Supplementary Material S6**.

### 2.8 Statistical analysis

In this study, model performance was evaluated using metrics including accuracy, sensitivity, specificity, positive predictive value (PPV), negative predictive value (NPV), and area under the receiver operating characteristic curve (AUC). Comparing these metrics aids in assessing the model’s classification capability, accuracy, and reliability. The formulas for calculating classification performance metrics are provided in Electronic **Supplementary Material S7**.

Net reclassification improvement (NRI) is a metric used to evaluate the improvement of a predictive model, particularly for comparing the performance differences between two models in a classification task. The formula for calculating the metrics is provided in Electronic **Supplementary Material S8**.

Decision curve analysis (DCA) is a method for evaluating the performance of medical diagnostic or predictive models. The primary objective of DCA is to assess the impact of model classification results at different thresholds, assisting medical decision-makers in making more informed choices in various scenarios, thus enhancing overall patient benefit.

All statistical analyses were performed using Python 3.7 (https://www.python.org/), MATLAB R2020b (https://www.mathworks.com/products/matlab.html), and R 4.3.0 (http://www.rproject.org). The deep learning model was constructed using Python. Mann-Whitney U test and Maximum MRMR algorithm were computed and analyzed using Python. The ESBELM classifier was built using MATLAB for classification. The “pROC” package in RStudio was utilized to plot ROC curves, the NRI value was calculated used the “glm” package in RStudio.

## 3 Results

### 3.1 Performance analysis of different classifiers

In this experiment, we conducted a comparative analysis among ESBELM, Ensemble Random Forest (ERF), and Extreme Learning Machine (ELM) to validate the superiority of ESBELM. The experimental results can be found in **Tables 2**, **3**. On the test set, ESBELM achieved AUC values of 0.7011, 0.7011, and 0.6805 when using single-phase features PCP, AP, and PVP as inputs, respectively. Additionally, M-DAFM achieved an AUC value of 0.7839, all of which outperformed the predictive performance of ERF and ELM.

**Table 2:** AUC values of different classifiers on the training set.

Classifier Input features	ERF	ELM	ESBELM
*PCP*	*0.6476*	*0.6865*	***0.7167***
*AP*	*0.5641*	*0.7170*	***0.7420***
*PVP*	*0.6229*	*0.6788*	***0.7045***
***DAFF***	*0.6877*	*0.7423*	***0.8013***

PCP, AP and PVP correspond to the features extracted from these three models, and DAFF represents domain-adaptive fused features.

The bold values are highlighted to emphasize the superiority of the classifier used in this study compared to other classifiers. The bolding of DAFF is intended to highlight the fused features obtained by the algorithm proposed in this paper.

**Table 3:** AUC values of different classifiers on the test set.

Classifier Input feature	ERF	ELM	SBELM
*PCP*	*0.6000*	*0.6621*	***0,7011***
*AP*	*0.5333*	*0.6920*	***0.7011***
*PVP*	*0.5828*	*0.6575*	***0.6805***
***DAFF***	*0.6460*	*0.7011*	***0.7839***

PCP, AP and PVP correspond to the features extracted from these three models, and DAFF represents domain-adapted fused features.

The bold values are highlighted to emphasize the superiority of the classifier used in this study compared to other classifiers. The bolding of DAFF is intended to highlight the fused features obtained by the algorithm proposed in this paper.

### 3.2 Analyzing the predictive performance of different models

We will compare the proposed M-DAFM model with the following models: (1) Single-modal model: Construct a deep learning model using only one phase of CT images (PCP, AP, or PVP) from patients for preoperative prediction of MVI; (2) Simple concatenation model (SC): Employ deep learning methods to extract deep features from PCP, AP, and PVP phase CT images of patients separately, followed by straightforward concatenation for preoperative prediction of MVI.; (3) State-of-the-Art models, where we selected two state-of-the-art image fusion models: TMC (Trusted Multi-View Classification model) (32), which dynamically acquires the credibility of different modalities and integrates information from each modality based on its credibility, thereby effectively improving the predictive performance of the model; CPM (Cross Partial Multi-View Networks) (33), which integrates information from different modalities by constructing a non-parametric classification loss function, allowing the model to learn the consistency and complementary information of different modalities for the target task, thereby enhancing model performance.

**Figure 2:**
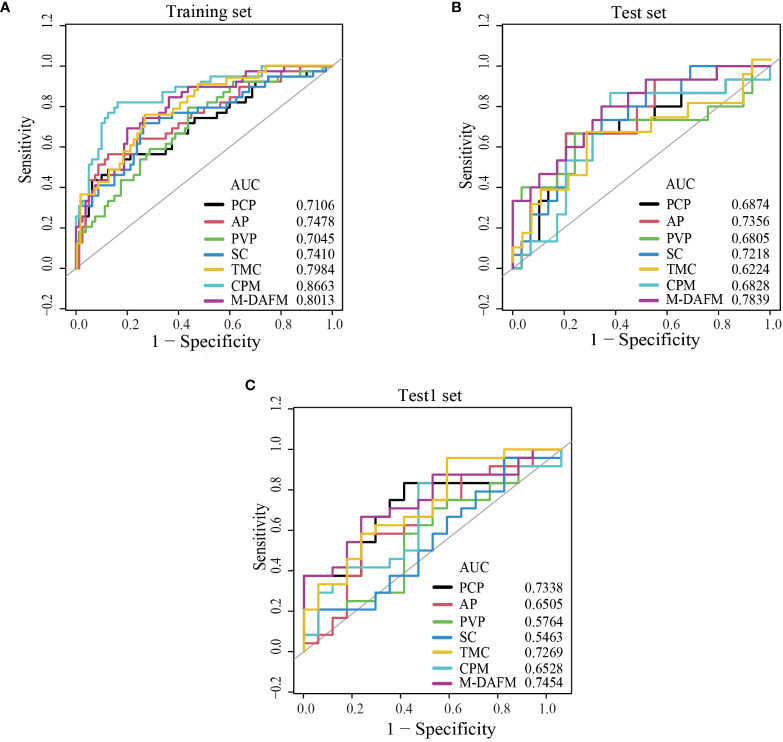
ROC curves of PCP, AP, PVP, SC, TMC, CPM models, and M-DAFM on the training **(A)**, test **(B)** and test1 **(C)** sets. SC, simple concatenation; TMC, trusted multi-view classification model; CPM, cross partial multi-view model. M-DAFM, multi-modal domain adaptive fusion model.

**Figure 3:**
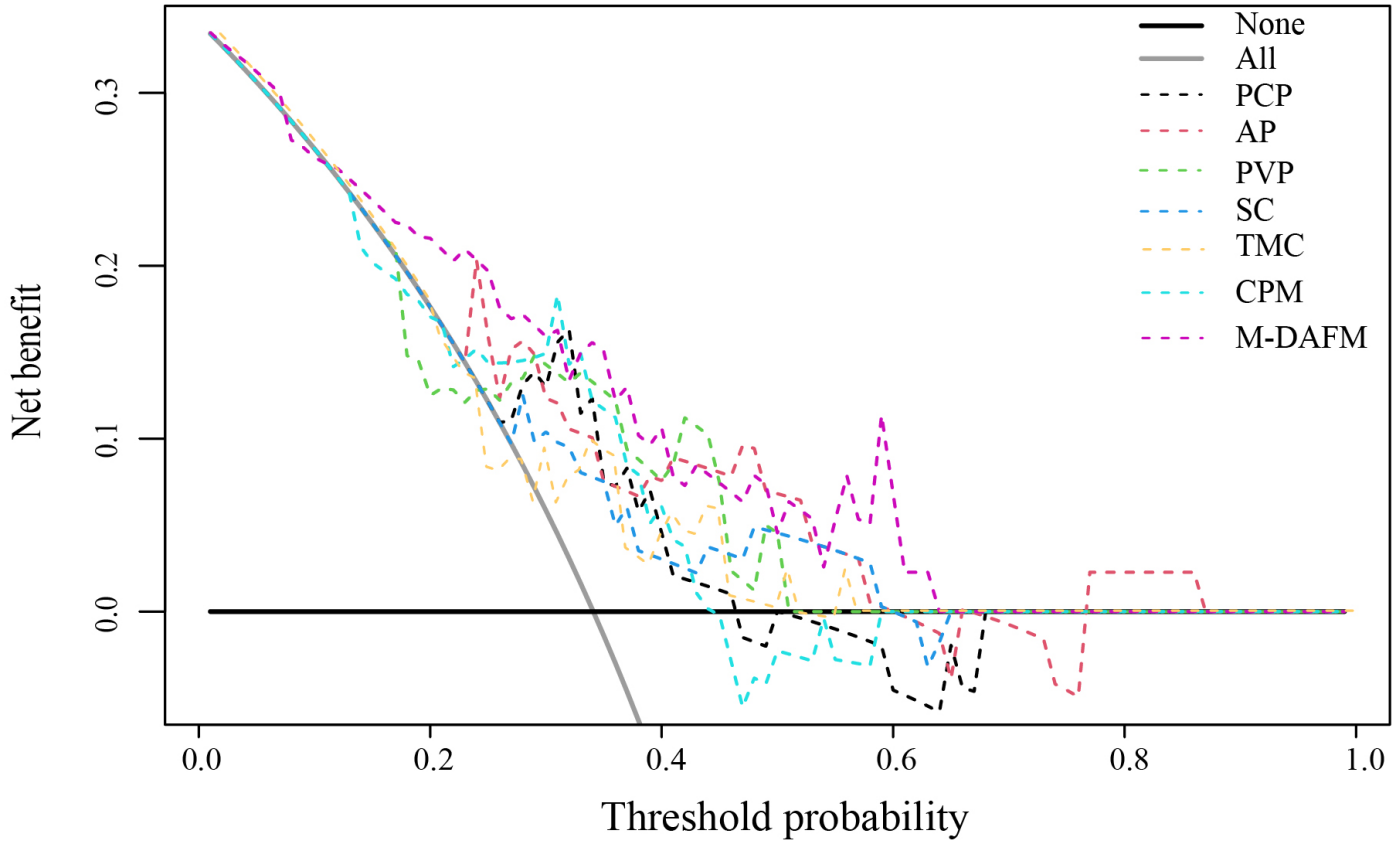
Decision curve analysis (DCA) of PCP, AP, PVP, SC, TMC, CPM models, and M-DAFM on the test set. SC, simple concatenation; TMC, trusted multi-view classification model; CPM, cross partial multi-view model. M-DAFM, multi-modal domain adaptive fusion model.

**Table 4** presents the diagnostic performance of each model for preoperative prediction of MVI; **Figure 2** shows the ROC (receiver operating characteristic) curves of each model on the training set, test set, and test1 set; **Table 5** demonstrates the improvement performance of M-DAFM compared to other models; **Figure 3** displays the DCA curves of each model on the test set.

**Table 4:** Comparison of classification performance between M-DAFM and PCP, AP, PVP, TMC, CPM and SC models.

Sets	Model	AUC	Sensitivity	Specificity	Accuracy	PPV	NPV
*Training Set*	*PCP*	*0.7106*	*0.4359 (17/39)*	*0.9375 (75/80)*	*0.7731 (92/119)*	*0.7727 (17/22)*	*0.7732 (75/97)*
	*AP*	*0.7478*	*0.5641 (22/39)*	*0.8750 (70/80)*	*0.7731 (92/119)*	*0.6875 (22/32)*	*0.8046 (70/87)*
	*PVP*	*0.7045*	*0.7949 (31/39)*	*0.5625 (45/80)*	*0.6387 (76/119)*	*0.4697 (31/66)*	*0.8491 (45/53)*
	*SC*	*0.7410*	*0.7179 (28/39)*	*0.7375 (59/80)*	*0.7311 (87/119)*	*0.5714 (28/49)*	*0.8429 (59/70)*
	*TMC*	*0.7984*	*0.7576 (25/33)*	*0.7361 (53/72)*	*0.7429 (78/105)*	*0.5682 (25/44)*	*0.8689 (53/61)*
	*CPM*	*0.8663*	*0.8205 (32/39)*	*0.8375 (67/80)*	*0.8319 (99/119)*	*0.7111 (32/45)*	*0.9054 (67/74)*
	***M-DAFM***	*0.8013*	*0.6923 (27/39)*	*0.8000 (64/80)*	*0.7647 (91/119)*	*0.6279 (27/43)*	*0.8421 (64/76)*
*Test set*	*PCP*	*0.6874*	*0.6667 (10/15)*	*0.7931 (23/29)*	*0.7500 (33/44)*	*0.6250 (10/16)*	*0.8214 (23/28)*
	*AP*	*0.7356*	*0.6667 (10/15)*	*0.7931 (23/29)*	*0.7500 (33/44)*	*0.6250 (10/16)*	*0.8214 (23/28)*
	*PVP*	*0.6805*	*0.6667 (10/15)*	*0.7586 (22/29)*	*0.7273 (32/44)*	*0.5882 (10/17)*	*0.8148 (22/27)*
	*SC*	*0.7218*	*0.7333 (11/15)*	*0.6552 (19/29)*	*0.6818 (30/44)*	*0.5238 (11/21)*	*0.8261 (19/23)*
	*TMC*	*0.6224*	*0.6429 (9/14)*	*0.7143 (20/28)*	*0.6905 (29/42)*	*0.5294 (9/17)*	*0.8000 (20/25)*
	*CPM*	*0.6828*	*0.8667 (13/15)*	*0.6207 (18/29)*	*0.7045 (31/44)*	*0.5417 (13/24)*	*0.9000 (18/20)*
	***M-DAFM***	*0.7839*	*0.8000 (12/15)*	*0.6552 (19/29)*	*0.7045 (31/44)*	*0.5455 (12/22)*	*0.8636 (19/22)*
*Test1 set*	*PCP*	*0.7338*	*0.8333 (20/24)*	*0.6111 (11/18)*	*0.7381 (31/42)*	*0.7407 (20/27)*	*0.7333 (11/15)*
	*AP*	*0.6505*	*0.5833 (14/24)*	*0.7778 (14/18)*	*0.6667 (28/42)*	*0.7778 (14/18)*	*0.5833 (14/24)*
	*PVP*	*0.5764*	*0.7083 (17/24)*	*0.5000 (9/18)*	*0.6190 (26/42)*	*0.6538 (17/26)*	*0.5625 (9/16)*
	*SC*	*0.5463*	*0.9583 (23/24)*	*0.2222 (4/18)*	*0.6429 (27/42)*	*0.6216 (23/37)*	*0.8000 (4/5)*
	*TMC*	*0.7269*	*0.9583 (23/24)*	*0.4444 (8/18)*	*0.7381 (31/42)*	*0.6970 (23/33)*	*0.8889 (8/9)*
	*CPM*	*0.6528*	*0.8333 (20/24)*	*0.5556 (10/18)*	*0.7143 (30/42)*	*0.7143 (20/28)*	*0.7143 (10/14)*
	***M-DAFM***	*0.7454*	*0.6667 (16/24)*	*0.7778 (14/18)*	*0.7143 (30/42)*	*0.8000 (16/20)*	*0.6364 (14/22)*

SC, simple concatenation; TMC, trusted multi-view classification model; CPM, cross partial multi-view model; M-DAFM, multi-modal domain adaptive fusion model.

**Table 5:** NRI comparison of M-DAFM with AP, PCP, PVP, TMC, CPM and SC models in the test set.

Model 1 Model 2		All-phase
*M-DAFM*	*PCP*	*NRI [95% CI]: 0.4805 [0.1393 - 0.8216] –P<0.05*
	*AP*	*NRI [95% CI]: 0.3471 [0.006 - 0.6882] – P<0.05*
	*PVP*	*NRI [95% CI]: 0.5379 [0.1416 - 0.9342] –P<0.05*
	*SC*	*NRI [95% CI]: 0.3816 [0.0396 - 0.7237] –P<0.05*
	*TMC*	*NRI [95% CI]: 0.1556 [-0.2049 - 0.516] –P=0.39*
	*CPM*	*NRI [95% CI]: 0.4092 [0.0798 - 0.7386] –P<0.05*

SC, simple concatenation; TMC, trusted multi-view classification model; CPM, cross partial multi-view model; M-DAFM, multi-modal domain adaptive fusion model.

As evident from **Table 4** and **Figure 2**, M-DAFM achieved AUC values in the test set close to those in the training set (<5%). This indicates that M-DAFM successfully learned relevant and effective information highly correlated with the target task in the training set. Moreover, M-DAFM exhibited good predictive performance on the test1 set with an AUC of 0.7454, indicating strong generalization capabilities. In other words, the model performed well on datasets with substantial differences from the training set. In comparison, the AUC values of single-modal models (PCP, AP, PVP) and the SC were consistently lower than M-DAFM across all three datasets. The AUC values of TMC and CPM in the test set are significantly lower than those in the training set, indicating a certain degree of overfitting. This implies that both TMC and CPM models have overly adapted to the noise or specific characteristics of the training set during the training process, leading to suboptimal performance on unseen data. Therefore M-DAFM exhibits superior predictive and generalization performance compared to other models, while TMC and CPM require further optimization to enhance their generalization performance on unknown data.

According to **Table 5**, the NRI values of M-DAFM compared to the single-modal models (PCP, AP, PVP) and the SC are 0.4805 (p < 0.05), 0.3471 (p < 0.05), 0.5379 (p < 0.05), and 0.3816 (p < 0.05), respectively. This indicates that M-DAFM exhibits a significantly improved predictive performance compared to these models. Furthermore, the NRI values of M-DAFM compared to the current state-of-the-art models, TMC and CPM, are 0.1556 (p = 0.39) and 0.4092 (p < 0.05), respectively, suggesting that M-DAFM still demonstrates some improvement in predictive performance compared to the current state-of-the-art models.

According to **Figure 3**, we visually represented the DCA curves for all models in the test set. It can be observed that, within the 0-0.65 threshold range, M-DAFM consistently achieves better or the best net benefit compared to other models.

In summary, through quantitative visual comparisons and analyses from various perspectives, including AUC, NRI, and DCA, we found that M-DAFM demonstrates excellent performance in preoperative prediction of MVI. Based on these analytical results, it can be concluded that M-DAFM not only excels in predictive performance but also holds significant potential for clinical applications.

## 4 Discussion

The diagnosis of MVI can only be confirmed through postoperative pathological examination, while the preoperative diagnosis of MVI relies on liver biopsy (34). However, due to factors such as tumor heterogeneity and challenges in sample acquisition, preoperative liver biopsy faces several limitations (35). If it were possible to predict the status of MVI preoperatively, doctors could tailor personalized treatment plans for patients at an earlier stage, thereby improving patient survival rates.

With the application of deep learning in the medical field, there have been studies that use deep learning methods to construct deep models for the preoperative prediction of MVI (22–24). In clinical practice, CT images at different phases can reveal the tumor’s vascular characteristics and its surrounding relationships at different time points. PCP images primarily display the basic anatomical features of the liver; AP images significantly enhance the detection of hepatic arterial blood flow, and PVP images can detect the blood flow and vascular distribution in the portal vein of the liver. Therefore, finding an objective and efficient way to integrate multi-phase image information, complementing the characteristics of each phase, may prove effective for diagnosis. This study innovatively predicts MVI by constructing the M-DAFM, which combines effective information from PCP, AP, and PVP modalities. Experimental validation demonstrates the superiority of multi-modal image fusion.

Comparative experiments with different classifiers reveal, as shown in **Tables 2**, **3**, that ESBELM performs the best in classifying MVI. This is possibly because CT image data often contain complex features and non-linear relationships, such as tumor morphology, texture, and vascular distribution. In contrast, ERF is insensitive to complex non-linear relationships, ELM is prone to overfitting when dealing with complex data, while ESBELM, by introducing ensemble strategies and Bayesian optimization algorithms, enhances its ability to handle high-dimensional and non-linear relationships while mitigating model overfitting.

Comparative experiments between single-modal models and multi-modal fusion models: As shown in **Figure 2** and **Table 4**, M-DAFM demonstrates superior performance in preoperative MVI prediction (The AUC values for the training set, test set, and Test1 set are 0.8013, 0.7839, and 0.7454, respectively). This could be attributed to the successful reduction of inter-modal differences by M-DAFM, allowing the model to better leverage complementary information from each modality for preoperative MVI prediction. In contrast, the performance of single-modal models (PCP, AP, PVP) in this aspect is significantly lower than that of M-DAFM, possibly due to the limited effective information provided by a single CT modality image, restricting the performance of single-modal models in preoperative MVI prediction tasks.On the other hand, the performance of SC in preoperative MVI prediction is relatively average, and even its predictive performance in the test set and Test1 set is inferior to some single-modal models. This may be because each modality typically predicts MVI from different perspectives, and SC does not consider the correlation between modalities, leading to negative interactions between modalities and affecting the predictive performance of SC.Regarding TMC and CPM, although they also integrate information from multiple modalities, the strategies adopted by these models may struggle to effectively distinguish between valuable information and noise within CT images, which often contain rich and complex microscopic information, encompassing multi-level structures of tumor lesions. This difficulty in effective discrimination may result in suboptimal predictive performance for these models.

The method proposed in this paper for preoperative prediction of MVI has three advantages in clinical practice:1) In terms of tumor segmentation, we employ a semi-automatic segmentation algorithm that only requires radiologists to perform rough segmentation of the tumor area. This significantly reduces the workload for radiologists in tumor segmentation, while reducing subjective interventions during the segmentation process. Consequently, it enhances the consistency and repeatability of the final results.2) Regarding feature extraction, we utilize a convolutional neural network for automatic, accurate, and objective extraction of specific features from the tumor region.3) In clinical practice, doctors often employ various methods for disease diagnosis. Inspired by this, our study considers the PCP, AP, and PVP of CT images as three distinct modalities. Using a domain adaptation approach, we design a multimodal fusion network to build a more robust and accurate preoperative prediction model, which holds practical significance.

This retrospective study has certain limitations. Firstly, the extensive time span of data collection may introduce variations in data quality. However, our experimental results demonstrate the effectiveness of the proposed method, highlighting the robustness of M-DAFM. Further improvements in data quality may enhance the model’s performance. Secondly, this study lacks multi-center CT image data for further validation of the model’s universality. Lastly, this study only explores the diagnostic performance of deep learning models, which enhances practical portability but may compromise accuracy. As for analyzing clinical models as a single modality within the multi-modal fusion model, we will continue to investigate in our future research.

## 5 Conclusions

This study introduces a novel approach for preoperative MVI prediction by effectively integrating information from multi-phase CT images through mitigating the distribution differences between different modalities.

## Data availability statement

The original contributions presented in the study are included in the article/**Supplementary Material**. Further inquiries can be directed to ZY, 1023494842@qq.com.

## Ethics statement

The studies involving humans were approved by the institutional review board of Jiangmen Central Hospital, which waived the requirement for written informed consent for participation. The studies were conducted in accordance with the local legislation and institutional requirements.

## Author contributions

ZY: Conceptualization, Methodology, Writing – original draft. YL: Conceptualization, Methodology, Writing – review & editing. XD: Conceptualization, Supervision, Writing – review & editing. EC: Conceptualization, Data curation, Writing – review & editing. JC: Data curation, Investigation, Writing – review & editing. CM: Conceptualization, Data curation, Writing – review & editing.

## Glossary

**Table:**

CT	Computed tomography
PCP	Pre-contrast phase
AP	Arterial phase
PVP	Portal venous phase
CNN	Convolutional neural networks
ROI	Region of interest
MVI	Microvascular invasion
HCC	Hepatocellular carcinoma
M-DAFM	Multi-modal domain adaptive fusion model
SC	Simple connection
TMC	Trusted multi-view classification model
CPM	Cross partial multi-view model
MRMR	Maximum relevance minimum redundancy
MMD	Maximum mean discrepancy
NRI	Net reclassification improvement
PPV	Positive predictive value
NPV	Negative predictive value
ELM	Extreme learning machine
ERF	Ensemble random forest
ESBELM	Ensemble sparse Bayesian extreme learning machine
AUC	Area under the receiver operating characteristic curve
ROC	Receiver operating characteristic
DCA	Decision curve analysis
