# Supplementary material for: Enhancing preoperative diagnosis of microvascular invasion in hepatocellular carcinoma: domain-adaptation fusion of multi-phase CT images
Source: Front Oncol. 2024 Jan 25;14:1332188. doi: 10.3389/fonc.2024.1332188 (PMC10851167; doi:10.3389/fonc.2024.1332188)
Supplement: Supplementary file 1 [file DataSheet_1.docx]

**Electronic Supplementary Material S1: Inclusion and Exclusion Criteria.**

**
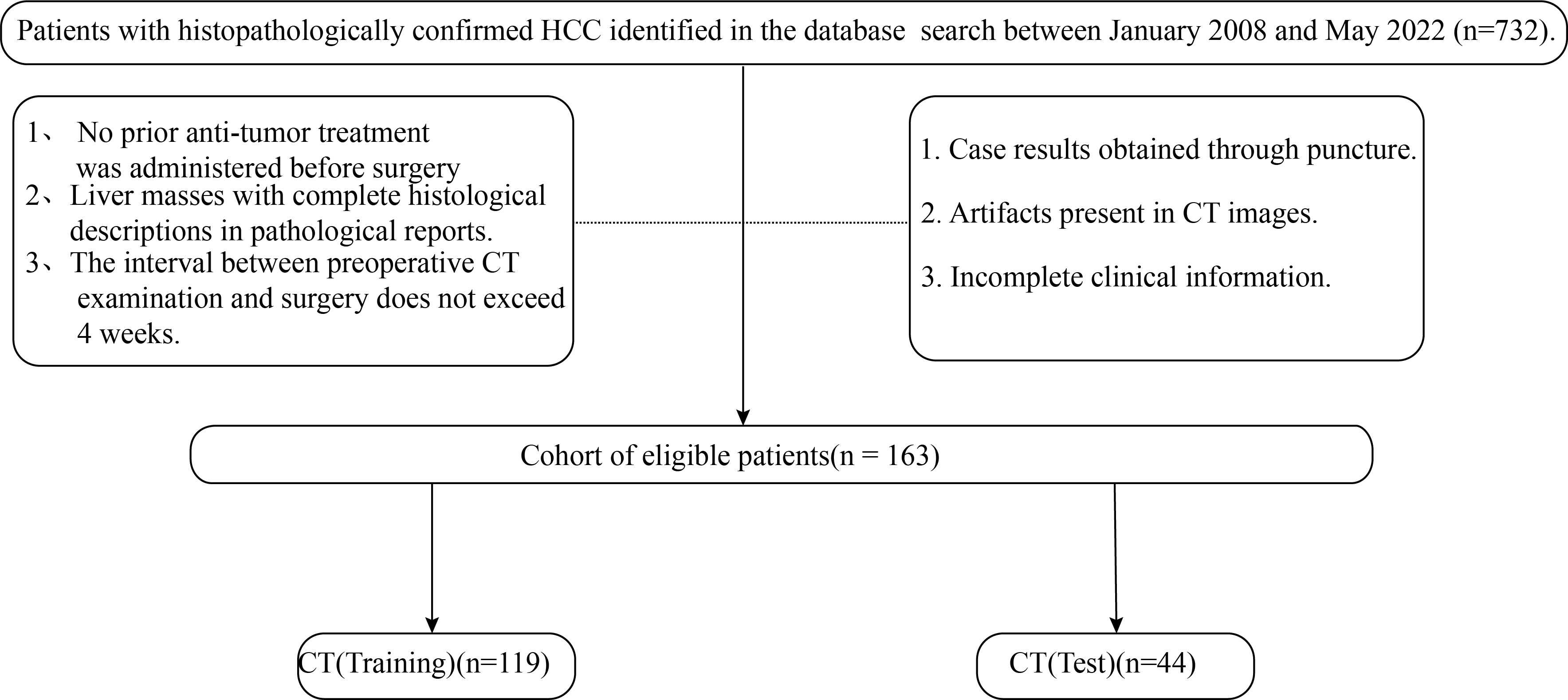
**

**Figure 1.** The patient inclusion and exclusion criteria

**Electronic Supplementary Material S2: Image Segmentation and Preprocessing**

To meet the requirements of training deep learning models, hepatocellular carcinoma grading images need to undergo preprocessing, as illustrated in Figure 2. Firstly, 3DSlicer software is used to annotate the liver image lesions from the starting to the ending slice, and during the annotation process, the lesion region is expanded by 10 millimeters to ensure complete coverage of the lesion area. Then, a Python-developed algorithm is utilized to extract the annotated lesion region, following a process that involves inscribing a circle within the annotated region to create a square, and extracting the area of this square. Finally, the extracted lesion images are normalized to a size of 224x224.


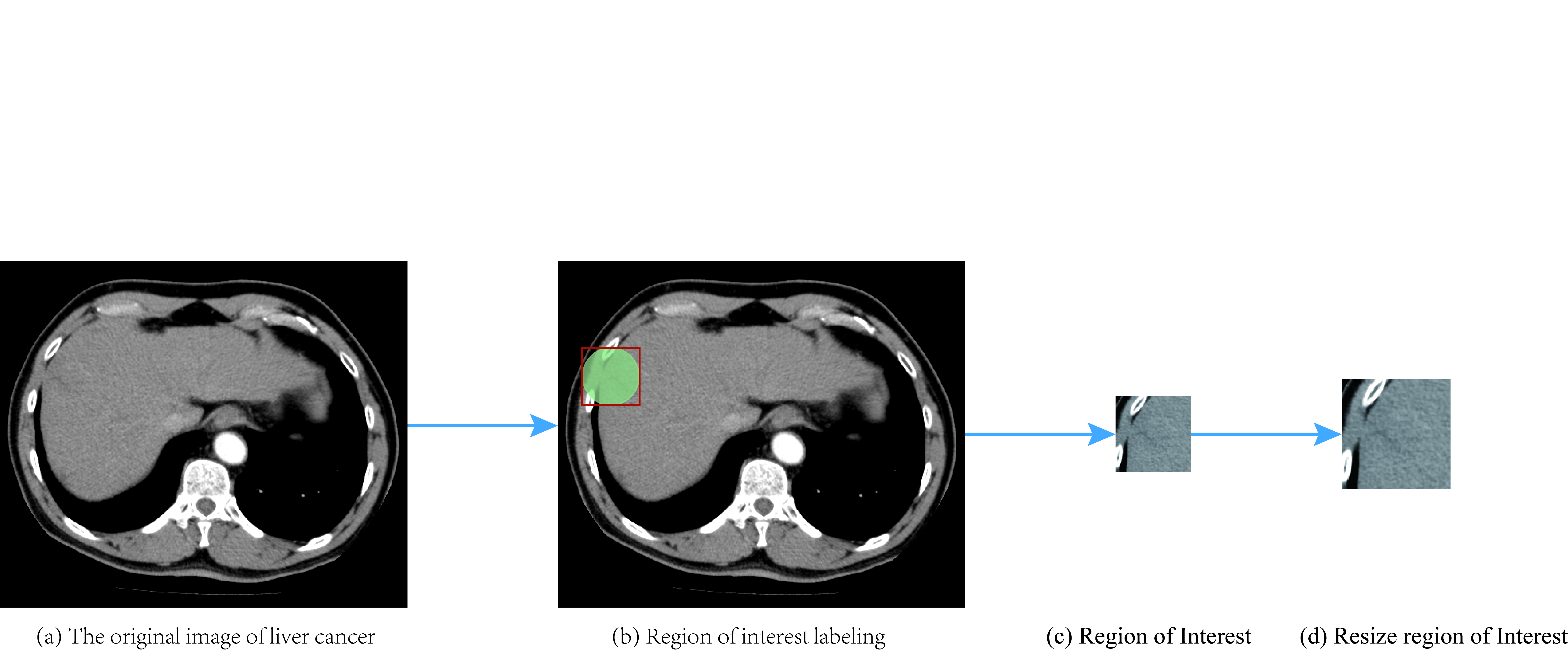


**Figure 2.** Hepatocellular carcinoma image preprocessing process

**Electronic Supplementary Material S3: Deep Learning Model Parameters**

The entire training process was implemented on a machine with an Intel Core i7-12700 CPU and an NVIDIA GeForce RTX 3060 Laptop GPU, using Python 3.7. The hyperparameter settings for the ResNet18 model are shown in Table 1.

**Table 1.** Model Hyperparameter Settings

| *Hyperparameter* | *Value* | *Hyperparameter* | *Value* |
| --- | --- | --- | --- |
| *Learning rate* | *0.000001* | *optimizer* | *SGD* |
| *Batch size* | *128* | *Momentum* | *0.9* |
| *weight decay* | $10^{-8}$ | *Epoch* | *100* |
| *Scheduler* | *CosineAnnealingWarmRestarts* | *Input_size* | *3×224×224* |
| *Loss function* | *cross_entropy* |  |  |

**Electronic Supplementary Material S4: Deep Feature Extraction**

This study employed a ResNet18 deep learning model pretrained on the ImageNet dataset to extract task-relevant image features, and its network architecture is illustrated in Figure 3. Initially, fine-tuning of the pretrained ResNet18 was performed using PCP's CT images. Subsequently, all convolutional layers of the fine-tuned model were utilized as feature extractors to extract features from CT images at different scales. The same procedure was applied to extract features from AP and PVP images. Each single-phase CT image yielded 3904 deep features. The feature extraction process is depicted in Figure 4.


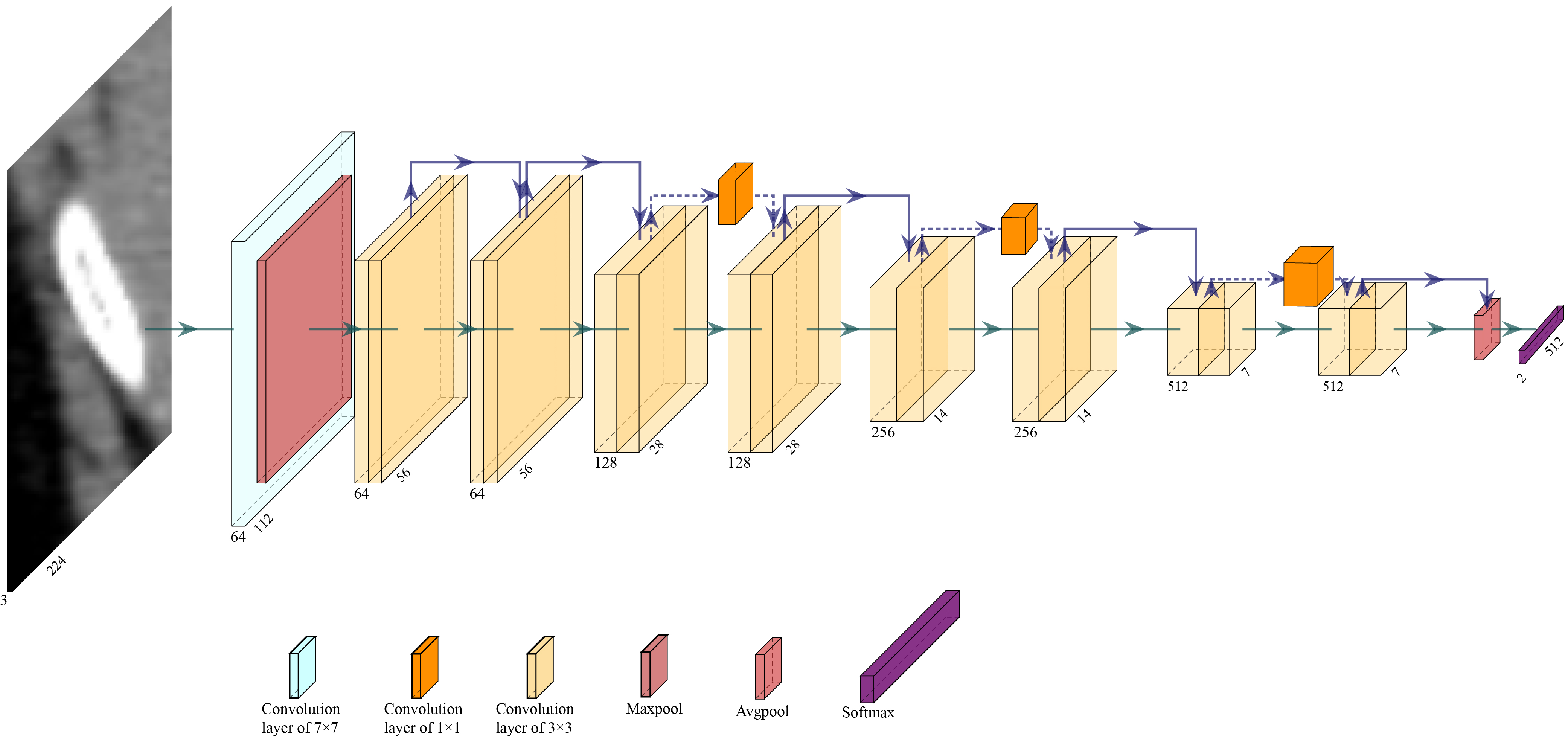


**Figure 3.** ResNet18 Network Architecture


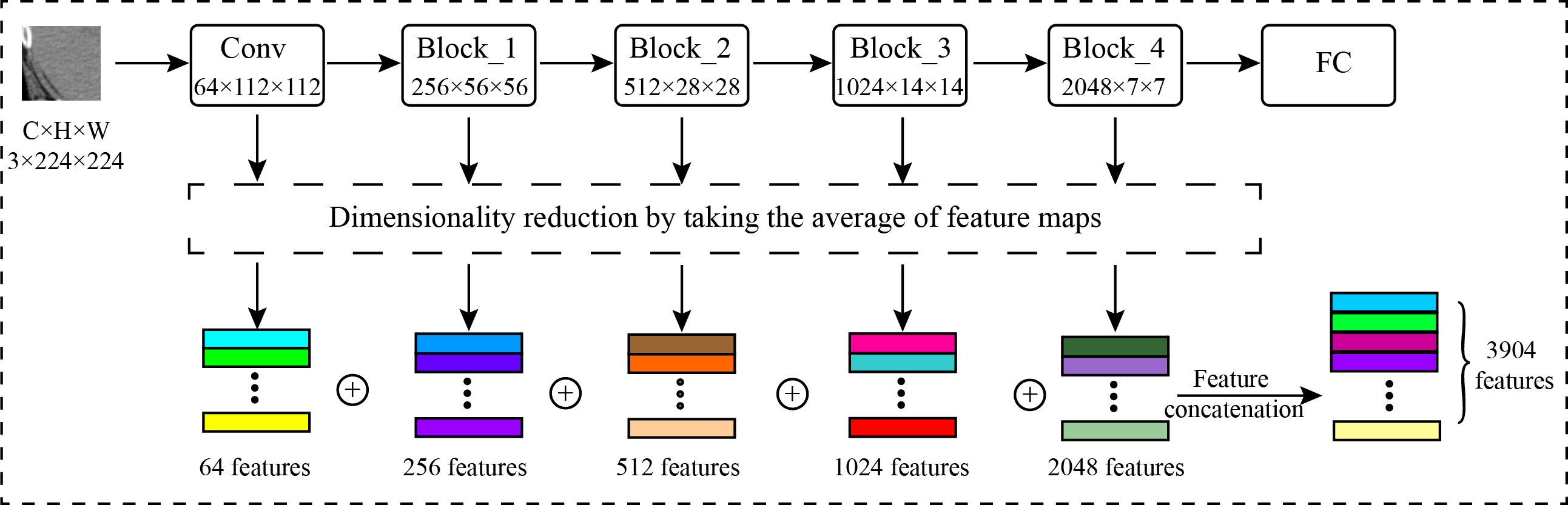


**Figure 4.** Feature Extraction Process

**Electronic Supplementary Material S5: Feature Fusion Process**

Due to the fact that PCP, AP, and PVP images reflect the vascular characteristics and spatial relationships of tumors at different time points, it is likely that there are differences in the feature distributions of CT images extracted by the deep model across various time periods. These differences can introduce some noise when performing feature concatenation, potentially leading to a reduction in the model's performance. Therefore, to address this issue, this study employed domain adaptation techniques based on transfer learning, utilizing Maximum Mean Discrepancy (MMD) to align the distributions of CT images from different time points. Subsequently, the features of CT images from different time periods were fused to obtain a more information-rich fusion model. The feature fusion process is illustrated in Figure 5.


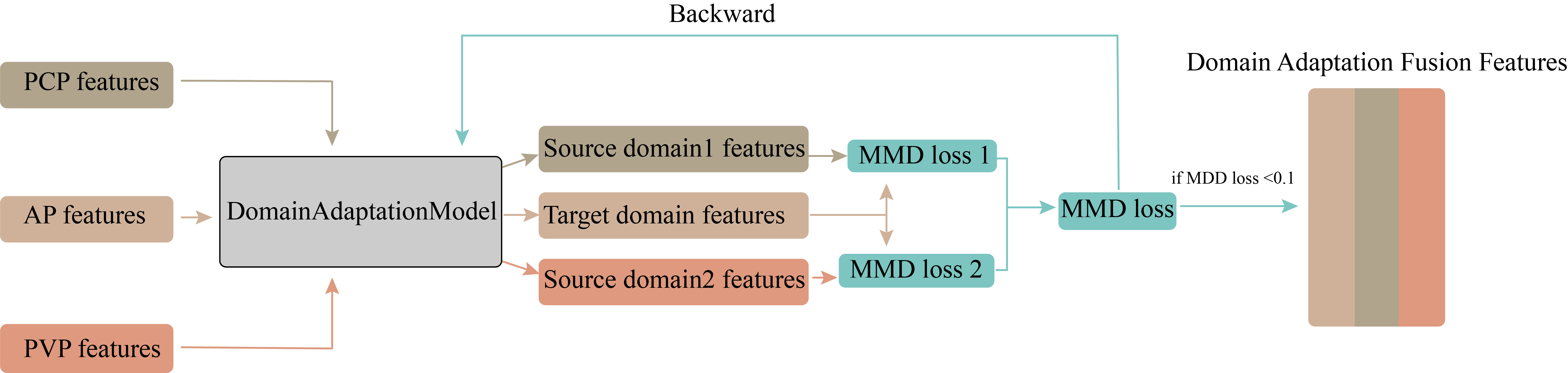


**Figure 5.** Domain-adaptive feature fusion

The definition of maximum mean discrepancy (MMD) is as follows:

(1)

Where $\{x_{i}^{s}\}_{i=1}^{m^{s}}$ represents the source domain dataset, and $\alpha$ denotes the marginal probability distribution in the source domain.$\{x_{j}^{t}\}_{j=1}^{m^{t}}$ stands for the target domain dataset, and $\beta$ represents the marginal probability distribution in the target domain. $\kappa$represents a reproducing kernel hilbert space (RKHS) represented by the feature kernel *K*，$m^{s}$stands for the number of samples in the source domain, and $m^{t}$ denotes the number of samples in the target domain. is a mapping function from the original space to the RKHS, and it satisfies the following relation: *k (x, y)* =, where *k (x, y)* represents a Gaussian kernel function, namely:

*k (x, y)* = *exp* ($\frac{-\left\|x-y\right\|}{\sigma^{2}}$) (2)

Where $\sigma$ represents the size of the Gaussian kernel. Combining formulas (1) and (2), the MMD between the source and target domains can be defined as:

$D=\frac{1}{(m^{s})^{2}}\sum_{i=1}^{m^{s}} \sum_{j=1}^{m^{s}} k(x_{i}^{s},x_{j}^{s})+\frac{1}{(m^{t})^{2}}\sum_{i=1}^{m^{t}} \sum_{j=1}^{m^{t}} k(x_{i}^{s},x_{j}^{s})-\frac{1}{m^{s}m^{t}}\sum_{i=1}^{m^{s}} \sum_{j=1}^{m^{t}} k(x_{i}^{s},x_{j}^{t})$ (3)

Thus, the domain-invariant representation between the target and source domains is learned through the Maximum Mean Discrepancy equation (3).

**Electronic Supplementary Material S6: Construction of ESBELM Classifier**

An ensemble classifier is created by combining multiple classifiers to achieve superior generalization performance compared to a single classifier. In this study, bootstrapping is employed to obtain training subsets that match the original data distribution. The Sparse Bayesian Extreme Learning Machine (SBELM) is selected as the base classifier to construct the ESBELM classifier, thereby enhancing the model's generalization performance. The workflow for constructing ESBELM is illustrated in Figure 6.


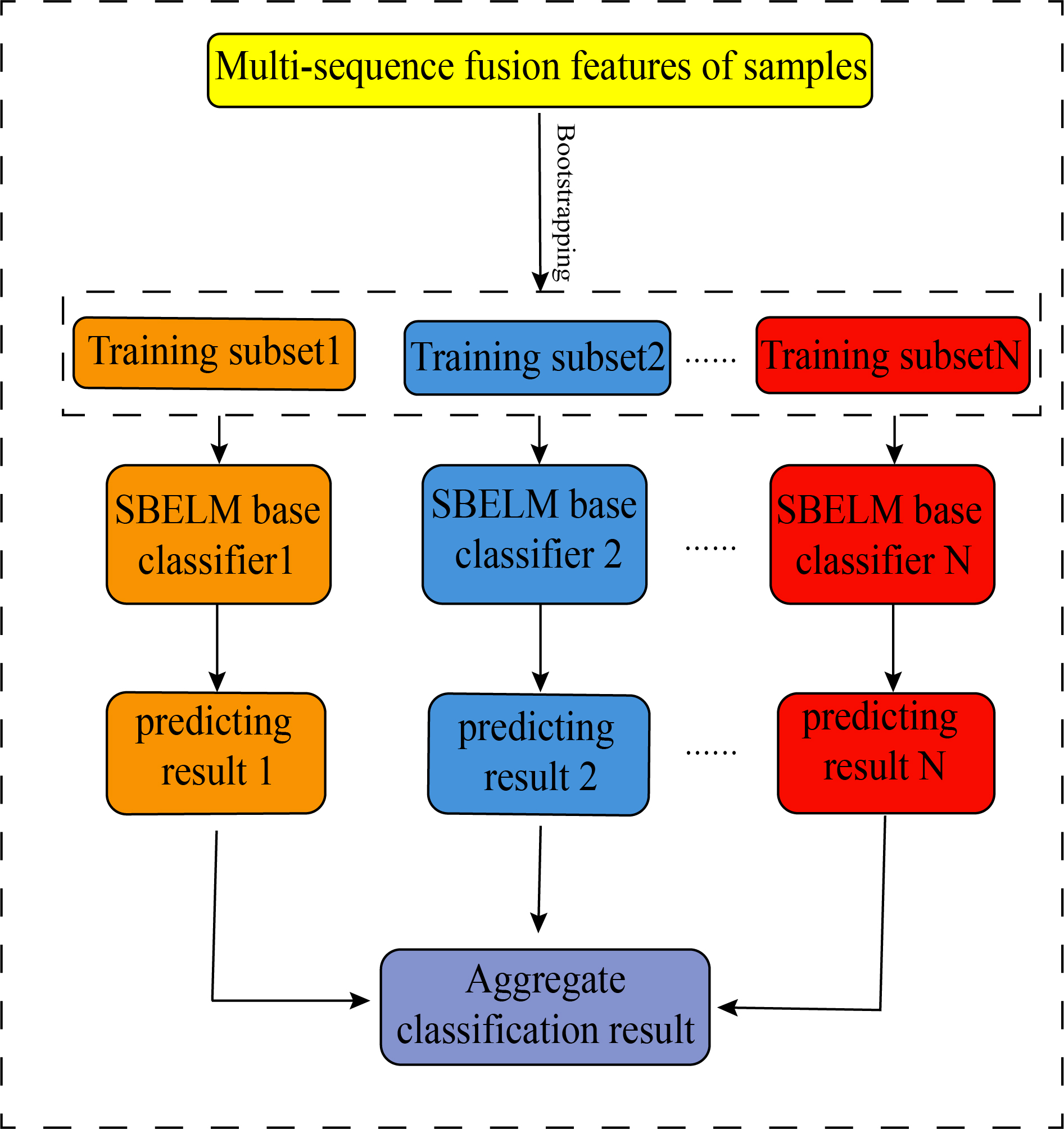


**Figure 6.** The workflow for constructing ESBELM

Extreme Learning Machine (ELM), proposed by Huang et al., is a single-hidden-layer feedforward neural network. Its main characteristic lies in requiring only the specification of the number of hidden layer neurons to randomly generate the weights and biases between the input and hidden layers. ELM demonstrates excellent performance in terms of learning speed and prediction accuracy.

The extreme learning machine employs sparse bayesian optimization for solution, using the bayesian linear regression algorithm to calculate the weights $LW$ and biases $b_{0}$ between the hidden and output layers of ELM. This algorithm models the weights using a prior distribution and constrains the weights during the model optimization process, encouraging less important feature weights to approach zero, thereby achieving sparsity.

$\mathrm{argmin}E(\omega,b)=\left\|y-X\omega\right\|^{2}+\lambda\sum_{i-1}^{L}\left\|\omega_{i}\right\|_{1}$ (4)

In the equation: *E* represents the expected loss; *ω* is the weight between the hidden and output layers; *b* is the bias between the hidden and output layers; *y* denotes the label value of the sample; λ>0 is the coefficient of the $L_{1}$ norm constraint term; *L* is the number of hidden layer neurons; *X* represents the output of the hidden layer.

The solution process of Equation (4) is as follows: For the sample data $\left\{x_{i},y_{i}\right\}_{i=1}^{N}$, where $X=\left(x_{1},x_{2},x_{3},...,x_{N}\right)^{T}$ is the hidden layer output matrix and $Y=(y_{1},y_{2},y_{3},...,y_{N})$ is the matrix of true labels. Let $\varepsilon\sim N(o,\sigma^{2})$ represent the noise. According to bayes' theorem, the posterior probability of $\omega$ is:

$P(\omega|X,Y)=\frac{P(Y|\omega,X)*P(\omega)}{P(Y|X)}=\frac{P(Y|\omega,X)*P(\omega)}{\int P(Y|\omega,X)*P(\omega)d\omega}$ (5)

$\int P(Y|\omega,X)*P(\omega)d\omega$ is the integration constant. The sparse prior probability of $\omega$ is taken as:

$P(\omega)=N(0,\text{ }\sum_{p})$ (6)

Where is the prior independent parameter for each output parameter $\omega_{i}$. The likelihood function:

$P(Y|\omega,X)=\prod_{i=1}^{N} P(y_{i}|\omega,x_{i})=\prod_{i=1}^{N} N(y_{i}|\omega^{T}x_{i},\sigma^{2})$ (7)

Utilizing the conjugacy property of the gaussian distribution, the posterior probability of $\omega$ is obtained as:

$P(\omega|X,Y)=N(\mu_{\omega},\text{∑}_{\omega})$ (8)

Where$\mu_{\omega}$ and can be represented as:

$\mu_{\omega}=\sigma^{-2}\text{∑}_{\omega}X^{T}Y$，$\text{∑}_{\omega}^{-1}=\sigma^{-2}X^{T}X+\text{∑}_{p}^{-1}$ (9)

$\sigma^{-2}$ and are solved using the method of marginal maximum likelihood estimation:

$\left\{\begin{matrix} \text{∑}_{pi}^{j+1}=\frac{1-\text{∑}_{pi}^{j}\text{∑}_{ii}}{\mu_{i}^{T}\mu_{i}} \\ (\sigma^{2})^{j+1}=\frac{\left\|Y-X\mu\right\|}{N-\sum_{i=1}^{L} (1-\text{∑}_{pk}^{j}\text{∑}_{kk})} \end{matrix}\right.$ (10)

In the equation, $\mu_{i}$ represents the i-th component of the posterior mean $\mu_{\omega}$, and represents the i-th diagonal component of the posterior distribution covariance matrix .

By initializing $\sigma^{-2}$ and , equation (9) is iteratively solved, yielding the gaussian distribution mean $\mu_{\omega}$ and covariance at maximum likelihood. If $\omega$ does not converge, parameters $\sigma^{-2}$ and are updated according to equation (10), and the mean $\mu_{\omega}$ and covariance are recalculated. If $\omega$ converges, then $\omega$=$\mu_{\omega}$. Due to some elements of approaching infinity during solving, the corresponding components of $\omega$ have posterior distributions of zero. The model is then constructed using the output $\omega$ of non-zero vectors for prediction.

In this study, bayesian linear regression algorithm is employed to determine the optimal weight values for base classifiers, which characterize the contribution of each base classifier in the final prediction. Subsequently, the predictions from individual base classifiers are combined by weighted summation, effectively integrating the predictions from multiple base classifiers to enhance the overall predictive performance and robustness of the model.

$T=(X\times L)+B$ (11)

In this context, *T* represents the final aggregated outcome, *X* denotes the predictions from all base classifiers, *L* stands for weights, and *B* represents bias.

**Electronic Supplementary Material S7: Formulas for classification performance metrics**

$\left\{\begin{matrix} Accuracy=\frac{TP+TN}{TP+TN+FP+FN} \\ Sensitivity=\frac{TP}{TP+FN} \\ Specificity=\frac{TN}{TN+FP} \\ PPV=\frac{TP}{TP+FP} \\ NPV=\frac{TN}{TN+FN} \end{matrix}\right.$ (12)

In equation (12), TP (true positive) represents the number of cases where MVI is positive and correctly detected as positive; FP (false positive) represents the number of cases where MVI is negative but incorrectly detected as positive; TN (true negative) represents the number of cases where MVI is negative and correctly detected as negative; FN (false negative) represents the number of cases where MVI is positive but incorrectly detected as negative.

**Electronic Supplementary Material S8: NRI (net reclassification improvement) index calculation formula**

$NRI=\frac{c_{1}-b_{1}}{N_{1}}+\frac{b_{2}-c_{2}}{N_{2}}$ (13)

In formula (13), where $N_{1}$ represents the total number of patients in the group, $c_{1}$ represents the number of individuals correctly classified by the new diagnostic indicator but incorrectly classified by the old diagnostic indicator, and $b_{1}$ represents the number of individuals incorrectly classified by the new diagnostic indicator but correctly classified by the old diagnostic indicator. In this context, the proportion of correct classifications improved by the new model relative to the old model is given by $\frac{c_{1}-b_{1}}{N_{1}}$. Similarly, for the non-patient group of $N_{2}$ individuals, the proportion of correct classifications improved by the new diagnostic model relative to the old diagnostic model is $\frac{b_{2}-c_{2}}{N_{2}}$.
